# Not all kidney cysts are created equal: a distinct renal cystogenic mechanism in tuberous sclerosis complex (TSC)

**DOI:** 10.3389/fphys.2023.1289388

**Published:** 2023-11-08

**Authors:** Manoocher Soleimani

**Affiliations:** ^1^ Department of Medicine, New Mexico Veterans Health Care Center, Albuquerque, NM, United States; ^2^ Department of Medicine, University of New Mexico School of Medicine, Albuquerque, NM, United States

**Keywords:** intercalated cells, kidney cysts, FOXI1, mTORC1, c-KIT, AVPR1A

## Abstract

Tuberous Sclerosis Complex (TSC) is an autosomal dominant genetic disease caused by mutations in either *TSC1* or *TSC2* genes. Approximately, two million individuals suffer from this disorder worldwide. TSC1 and TSC2 code for the proteins harmartin and tuberin, respectively, which form a complex that regulates the mechanistic target of rapamycin complex 1 (mTORC1) and prevents uncontrollable cell growth. In the kidney, TSC presents with the enlargement of benign tumors (angiomyolipomas) and cysts whose presence eventually causes kidney failure. The factors promoting cyst formation and tumor growth in TSC are poorly understood. Recent studies on kidney cysts in various mouse models of TSC, including mice with principal cell- or pericyte-specific inactivation of TSC1 or TSC2, have identified a unique cystogenic mechanism. These studies demonstrate the development of numerous cortical cysts that are predominantly comprised of hyperproliferating A-intercalated (A-IC) cells that express both TSC1 and TSC2. An analogous cellular phenotype in cystic epithelium is observed in both humans with TSC and in TSC2^+/−^ mice, confirming a similar kidney cystogenesis mechanism in TSC. This cellular phenotype profoundly contrasts with kidney cysts found in Autosomal Dominant Polycystic Kidney Disease (ADPKD), which do not show any notable evidence of A-IC cells participating in the cyst lining or expansion. RNA sequencing (RNA-Seq) and confirmatory expression studies demonstrate robust expression of Forkhead Box I1 (FOXI1) transcription factor and its downstream targets, including apical H^+^-ATPase and cytoplasmic carbonic anhydrase 2 (CAII), in the cyst epithelia of *Tsc1* (or *Tsc2*) knockout (KO) mice, but not in Polycystic Kidney Disease (*Pkd1*) mutant mice. Deletion of FOXI1, which is vital to H^+^-ATPase expression and intercalated (IC) cell viability, completely inhibited mTORC1 activation and abrogated the cyst burden in the kidneys of *Tsc1* KO mice. These results unequivocally demonstrate the critical role that FOXI1 and A-IC cells, along with H^+^-ATPase, play in TSC kidney cystogenesis. This review article will discuss the latest research into the causes of kidney cystogenesis in TSC with a focus on possible therapeutic options for this devastating disease.

## Introduction and discussion

TSC is caused by mutations in either TSC1 or TSC2 genes and affects multiple organs; including the kidney, heart, lung, and brain (Sampson and Harris, 1994; Crino et al., 2006; Henske et al., 2016; Rosset et al., 2017). TSC renal disease is characterized by the development and enlargement of cysts and benign tumors (angiomyolipomas) whose presence results in the decline of kidney function and eventually leads to end stage renal disease (Bissler et al., 2008; Henske et al., 2014; Bissler and Kingswood, 2018; Gallo-Bernal et al., 2022). The incidence of renal cell carcinoma (RCC) is increased in TSC, specifically affecting younger individuals afflicted with disease (Henske et al., 2016). Although the initiating event in TSC1 or TSC2 is well described, the factors that promote the kidney disease phenotype and progression are poorly understood.

## The role of mTORC1 in cyst and tumor growth in TSC kidneys

Loss of function of TSC1 or TSC2 activates mTORC1, a ubiquitous protein kinase complex that integrates systemic signals, such as growth factors and cytokines, with local signals that sense the availability of nutrients (e.g., amino acids, glucose and oxygen), in order to regulate cell growth (Urbanska et al., 2013; Kim and Guan, 2019). The mTORC1 activation is the principal mechanism driving growth in benign tumors (angiomyolipomas) and cystogenesis in TSC kidneys (Bissler et al., 2008; Henske et al., 2014; Bissler and Kingswood, 2018; Gallo-Bernal et al., 2022) by initiating transcriptional, translational, and post-translational processes that promote a multitude of anabolic activities; including inhibiting cellular catabolic processes resulting in cell growth and proliferation (Sarbassov et al., 2005; Düvel et al., 2010; Howell et al., 2013).

The mTORC1 is under the control of the TSC1-TSC2 complex and the Ras-Homolog Enriched in Brain (RHEB). RHEB plays a vital role in regulation of growth and cell proliferation through the insulin/mTOR/S6 Kinase (S6K) signaling pathway (Sarbassov et al., 2005; Düvel et al., 2010; Howell et al., 2013; Urbanska et al., 2013; Kim and Guan, 2019). The TSC1-TSC2 complex inhibits mTORC1 by negatively regulating RHEB-GTPase (Fingar and Blenis, 2004; Kwiatkowski and Manning, 2005; Sarbassov et al., 2005; Düvel et al., 2010; Howell et al., 2013; Henske et al., 2014; Bissler and Kingswood, 2018). In the presence of inactivating mutations in TSC1 or TSC2, RHEB-GTPase is no longer under the inhibitory control of TSC1/TSC2, and in its GTP-bound form can activate mTORC1, which then phosphorylates the downstream elements, S6K and the eIF4E-binding proteins (4-EBP), leading to cell proliferation and growth (Fingar and Blenis, 2004; Kwiatkowski and Manning, 2005; Sancak et al., 2010; Ögmundsdóttir et al., 2012; Showkat et al., 2014).

Angiomyolipomas are benign kidney tumors consisting of fat, muscle, and blood vessels. They are usually accompanied by cysts and are subject to progressive growth and hemorrhage (Bissler et al., 2008; Henske et al., 2014; Bissler and Kingswood, 2018; Gallo-Bernal et al., 2022). Because of robust mTORC1 activation and its role in cell proliferation, mTORC1 inhibitors (e.g., sirolimus or everolimus) have been used in the treatment of kidney disease in TSC patients (Bissler et al., 2008; Henske et al., 2014). Unfortunately, a significant portion of individuals with TSC do not respond to this therapy. Further, kidney lesions can return to their baseline size when drugs are discontinued (Bissler et al., 2019a).

## Loss of heterozygosity in TSC gene in angiomyolipomas vs. cysts in TSC kidney

Kidney cysts are usually composed of cells that express intact TSC1 and TSC2 proteins in both mouse models, as well as in humans with TSC renal cystic disease (Onda et al., 1999; Bonsib et al., 2016). This occurrence is distinct from the angiomyolipomas which show a loss of TSC gene and function (Bonsib et al., 2016; Giannikou et al., 2016). These results may suggest that the factors and pathways that promote cyst growth and expansion are unique and different from those promoting the formation of angiomyolipomas in the kidneys of individuals with TSC.

While expressing phenotypically distinct cell types lining the cysts, both ADPKD and TSC kidney cysts display mTORC1 activation (Shillingford et al., 2006; Henske et al., 2014; Bissler and Kingswood, 2018). Yet, the role of mTORC1 activation in ADPKD cystogenesis remains conflicting. In humans with TSC, inhibition of mTORC1 or S6K profoundly blunts the overgrowth of cells, tumors, and cysts in the kidney, as long as patients remain on these inhibitors (Bissler et al., 2008; Henske et al., 2014; Bissler and Kingswood, 2018). In contrast, the inhibition of mTORC1 in humans with ADPKD did not exhibit a significant beneficial impact on kidney function and cyst volume (Serra et al., 2010). These results display contrasting effects of mTORC1 in cystogenesis in TSC vs. ADPKD. With the exception of mTORC1 inhibitors, there are no therapeutic (druggable) molecular targets to alleviate kidney cysts or tumors in TSC.

## Distinct cell phenotype in cyst epithelia in TSC

The first studies examining the identity of cells lining the cysts in TSC came from heterozygote *Tsc2* (*Tsc2* ^+/−^) mice, which showed a predominance of IC cells within the cyst epithelia (Onda et al., 1999). A more recent report on *Tsc1* inactivation in mouse principal cells showed that kidney cysts exhibited a gradual loss of Aquaporin 2 (AQP-2)-positive cells in their epithelium (Chen et al., 2014). The disappearance of AQP-2-positive cells was attributed to the dedifferentiation of principal cells (Chen et al., 2014). However, there were no detailed characterizations of the non–AQP-2–expressing cells lining the cysts (Chen et al., 2014). Recently, we showed that in mice with either *Tsc1* or *Tsc2* deletion in kidney principal cells, the epithelia lining the cysts are predominantly comprised of cells exhibiting apical H^+^-ATPase and basolateral Cl^−^/HCO_3_^−^ exchanger AE1 (SLC4A1) (Bissler et al., 2019b; Barone et al., 2021; Zahedi et al., 2022; Barone et al., 2023). These results convincingly identified the cells lining the kidney cysts in TSC as A-IC cells (Bissler et al., 2019b; Barone et al., 2021; Zahedi et al., 2022; Barone et al., 2023) and demonstrate that the loss of AQP-2 positive cells was due to the disappearance of principal cells and their replacement with A-intercalated cells in the cyst lining.

In addition to the apical H^+^-ATPase and basolateral AE1 expression, cells lining the cysts expressed the proliferation marker Proliferating Cell Nuclear Antigen (PCNA), along with the intact TSC locus (Bissler et al., 2019b; Barone et al., 2021). These results strongly support the view that the cyst epithelial cells are hyperproliferating A-IC cells that are genotypically normal and express both TSC1 and TSC2 (Bissler et al., 2019b; Barone et al., 2021). A similar cell phenotype was observed in kidney cysts in mice with pericyte-specific inactivation of TSC1 (Bissler et al., 2019b; Barone et al., 2021). Furthermore, an identical cell phenotype was observed in the cystic epithelium of humans with TSC, confirming a similar TSC kidney cystogenesis mechanism (Bissler et al., 2019b; Barone et al., 2021). The epithelial cells lining the cysts in individuals with ADPKD and in mutant *Pkd*1 mouse models do not show any notable evidence of IC cell presence or their participation in cyst expansion (Holthöfer et al., 1990; Shibazaki et al., 2008).

**FIGURE 1:**
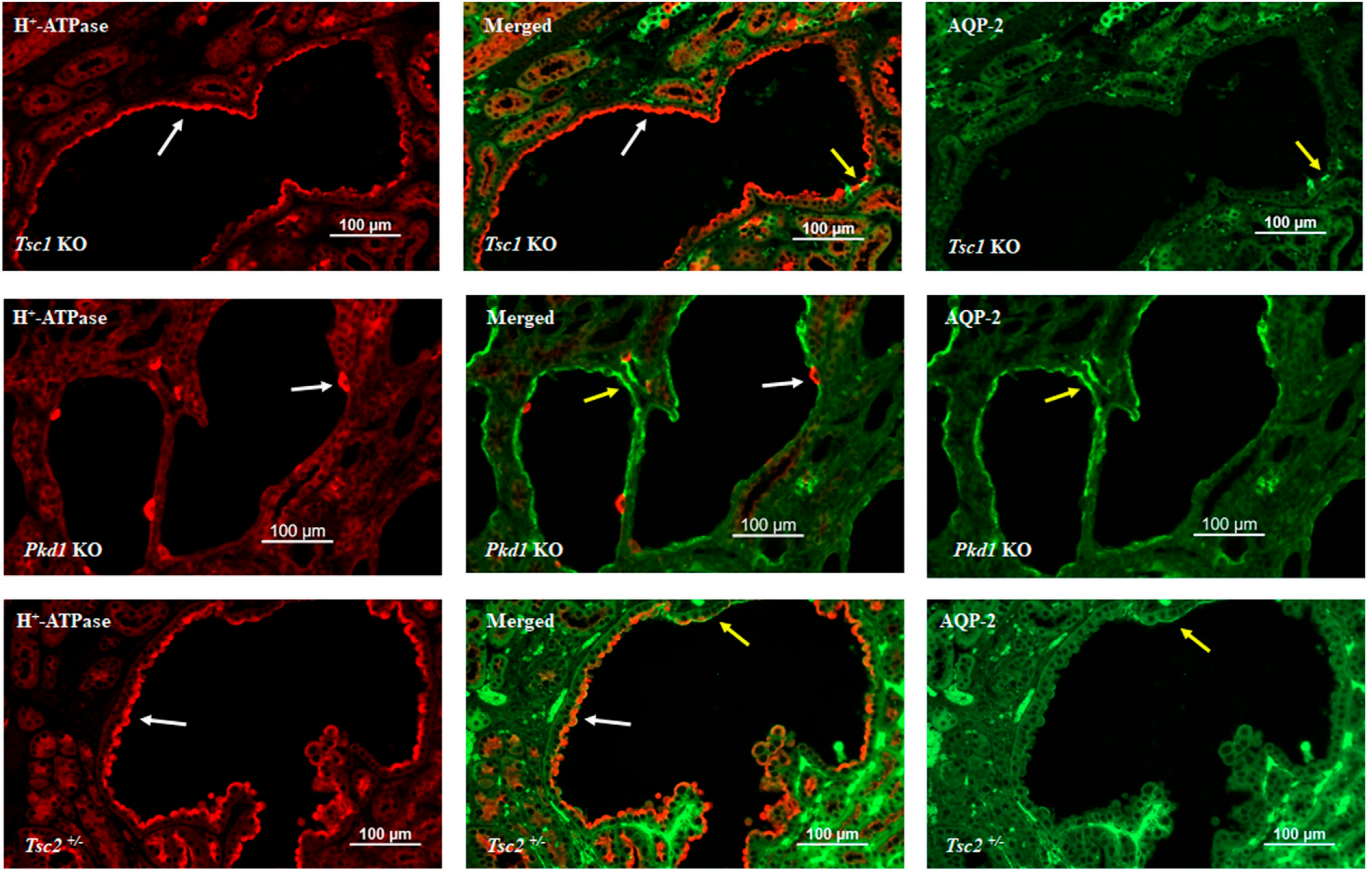
Double immunofluorescence labeling with H^+^-ATPase and AQP-2 in the kidney cysts of mice with principal cell-specific inactivation of *Tsc1* (top panel), principal cell-specific inactivation of *Pkd1* (middle panel), and *Tsc2* haploinsufficiency (Tsc2^+/−^). As indicated, H^+^-ATPase shows abundant and widespread expression on the apical membrane of cells lining the cysts, with very few AQP-2 labeling in *Tsc1* KO mice (top panel). The H^+^-ATPase expression in *Pkd1* mice is shown for comparison (middle panel) and indicates a completely different labeling pattern. There are few H^+^-ATPase positive cells lining the cysts; whereas, AQP-2 labeling is very robust in *Pkd1* cyst epithelia (middle panel). The expression of H^+^-ATPase was almost uniform in *Tsc2* ^+/−^ mice (bottom panel). Created with BioRender.com by Sharon Barone.

Figure 1 depicts double immunofluorescence labeling with H^+^-ATPase and AQP-2 in kidney cysts of mice with principal cell inactivation of *Tsc1* (top panel), kidney-specific inactivation of *Pkd1* (middle panel), and *Tsc2* haploinsufficiency (*Tsc2* ^+/−^). H^+^-ATPase shows abundant and widespread expression on the apical membrane of cells lining the cysts, with very few AQP-2 positive cells in *Tsc1* KO mice (top panel). The H^+^-ATPase expression in *Pkd1* mice is shown for comparison (middle panel) and indicates a completely different pattern, with few cells lining the cysts expressing H^+^-ATPase, whereas labeling with AQP-2 is very robust in cyst epithelia in *Pkd1* mutant mice (middle panel). The expression of H^+^-ATPase was almost uniform in *Tsc2* ^+/−^ mice (bottom panel). Please see legend to Figure 1.

## H^+^-ATPase and mTORC1 interaction: the essential role of the lysosome

The presence of apical H^+^-ATPase in cells lining the cysts has brought new attention to the role of this molecule in TSC renal cystic disease (Bissler et al., 2019b; Barone et al., 2021; Zahedi et al., 2022; Barone et al., 2023). Vacuolar H^+^-ATPase (V H^+^-ATPase) is a large, multi-subunit H^+^ pump composed of V0 (membrane-spanning) and V1 (catalytic) complexes, and couples the energy of ATP hydrolysis to H^+^ translocation across plasma and intracellular membranes (Forgac, 2007; Hinton et al., 2009). Examination of the role of V H^+^-ATPase has revealed a crucial role for this pump in luminal acidification in several intracellular organelles, including late endosomes and lysosomes (Forgac, 2007; Hinton et al., 2009). Essential to mTORC1 activation is its recruitment to the lysosomal surface, which has brought additional attention to this organelle as a signaling hub since it incorporates many contributing signals required for cell proliferation and growth (Sancak et al., 2010; Ögmundsdóttir et al., 2012; Showkat et al., 2014).

**FIGURE 2:**
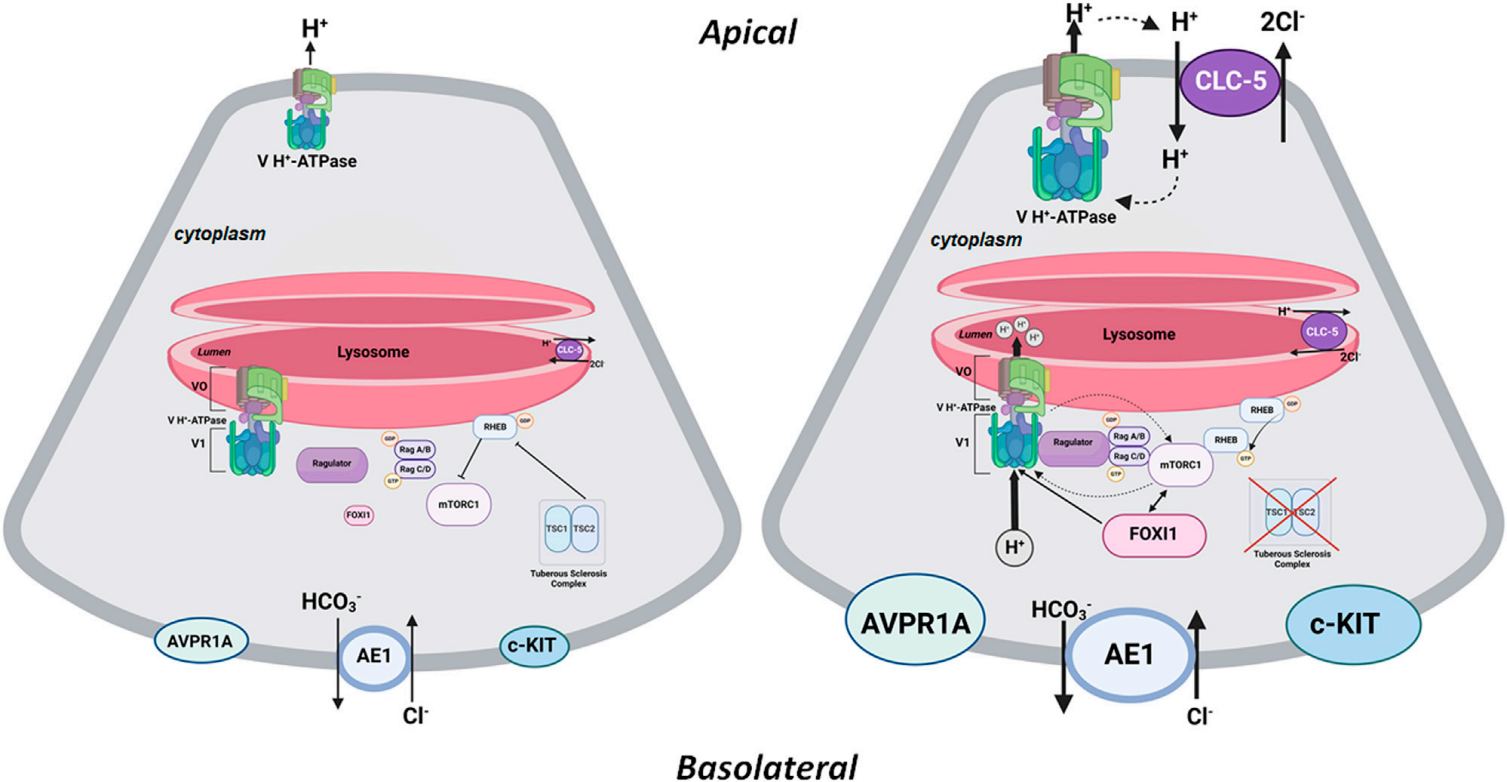
A schematic diagram depicting the interaction of mTORC1 and H^+^-ATPase on the lysosomal membrane. The left panel depicts a baseline state where the TSC1/TSC2 complex is intact and prevents RHEB from activating mTORC1. See additional description in the text under the heading, “The Central Role of FOXI1 in Kidney Cystogenesis in TSC.” The right panel depicts mTORC1 activation consequent to inactivating mutations or phosphorylation of TSC1 or TSC2. In its activated state, RHEB is released from the TSC1/TSC2 complex, resulting in the activation of mTORC1, which enhances the expression and assembly of V0 and V1 H^+^-ATPase subcomplexes (as depicted by bold arrows); therefore, resulting in increased H^+^-ATPase-mediate H^+^ transport. The feedback loop between mTORC1 and H^+^-ATPase also indicates the stimulatory effect of H^+^-ATPase on mTORC1 by enabling its recruitment and activation on the lysosomal membrane. In all mouse models of TSC examined thus far (Bissler et al., 2019b; Barone et al., 2021; Zahedi et al., 2022; Barone et al., 2023), mTORC1 is associated with enhanced expression of FOXI1 which activates H^+^-ATPase by enhancing the expression and activity of its subunits. Inhibition or inactivation of FOXI1 completely abrogates kidney cystogenesis and inhibits mTORC1 in TSC kidneys. The HCO_3_^−^ exit via the basolateral Cl^−^/HCO_3_^−^ exchange system (AE1; SLC4A1) is enhanced under an activated mTORC1 state. Created with BioRender.com by Sharon Barone.

Vacuolar H^+^-ATPase and mTORC1 have a reciprocal activating effect on each other in the lysosomal membrane. Recent studies have identified a network of signals/molecules that link mTORC1 to V H^+^-ATPase, a critical component of lysosomes. mTORC1 was able to enhance the expression of V H^+^-ATPases both in cells and mice (Peña-Llopis et al., 2011). The crosstalk between V H^+^-ATPase and mTORC1 on lysosome membranes is dependent on lysosomal biogenesis, which is regulated by Transcription factor EB (TFEB) (Peña-Llopis et al., 2011). Concurrently, increased H^+^-ATPase assembly and activity in the lysosomal membrane is necessary for amino acid-dependent mTORC1 recruitment (and activation) through interactions with the Rag GTPases, which are tethered to the lysosomal membrane by the Ragulator complex (Zoncu et al., 2011; Bar-Peled et al., 2013; Bar-Peled and Sabatini, 2014; Alesi et al., 2021). The inhibition or inactivation of H^+^-ATPase occurs through the decoupling of H^+^-ATPase from the Rag GTPases, leading to the inhibition of mTORC1 signaling (Forgac, 2007; Hinton et al., 2009; Nada et al., 2014). This inactivation leads to the neutralization of the luminal pH in lysosomes. It should be noted that the luminal lysosomal acidification consequent to the inward H^+^-ATPase-mediated H^+^ transport can simultaneously lead to the cytosolic alkalinization, which has further been implicated in the regulation of lysosomal perinuclear clustering (lysosomal topology) and mTORC1 (Chung et al., 2019). Taken together, these studies demonstrate the reciprocal stimulating effect of mTORC1 and H^+^-ATPase; mTORC1 enhances H^+^-ATPase expression and activity (Peña-Llopis et al., 2011; Alesi et al., 2021), while H^+^-ATPase plays a critical role in recruiting mTORC1 and sustaining its activation (Zoncu et al., 2011; Bar-Peled et al., 2013; Bar-Peled and Sabatini, 2014; Nada et al., 2014; Chung et al., 2019). Please see Figure 2 for more details.

## mTORC1 activation in cyst epithelium is mainly detected in H^+^-ATPase–overexpressing intercalated cells

The renal cyst epithelia in all mouse models of TSC display a robust mTORC1 activation as demonstrated by the presence of significantly elevated phospho-S6K levels (Bissler et al., 2019b; Barone et al., 2021). Active proliferation of A-IC cells in cyst epithelia was verified by a remarkable co-localization of PCNA and H^+^-ATPase in the same cells (Bissler et al., 2019b; Barone et al., 2021). These results indicate the activation of mTORC1 in A-IC cells lining the cysts and demonstrate that A-IC cells are the primary cells that are robustly proliferating in the epithelium of renal cysts.

## Vacuolar H^+^-ATPase in kidney A-IC cells: from intracellular organelles to the plasma membrane

V H^+^-ATPase is essential for the luminal acidification of intracellular organelles in all cells (Forgac, 2007; Hinton et al., 2009). This process includes acidifying the lumen of endosomes, lysosomes, and phagosomes; as well as several other intracellular organelles (Forgac, 2007; Hinton et al., 2009). In a few specialized cells, including kidney IC cells, pancreatic beta cells, and osteoclasts, V H^+^-ATPase plays two distinct roles. It pumps acid (H^+^) into the lumen of organelles, as well as across the plasma membrane into the extracellular compartment (Forgac, 2007; Hinton et al., 2009; Korolchuk et al., 2011; Soleimani and Rastegar, 2016; Do et al., 2022).

There is evidence supporting the recycling of V H^+^-ATPase between the plasma membrane and specialized intracellular vesicles (compartments) in kidney IC cells in response to either pH alteration or various stimuli, including several hormones, glucose, or signaling pathways (Carraro-Lacroix and Malnic, 2006; Brown et al., 2009; Alzamora et al., 2010; Gong et al., 2010; Ueno et al., 2014; Merkulova et al., 2015; Smith et al., 2016; Stransky et al., 2016; Eaton et al., 2021). One major pathway enhancing the trafficking of H^+^-ATPase from the specialized intracellular compartments to the plasma membrane is the activation of PKA by intracellular cAMP, which causes phosphorylation of several H^+^-ATPase subunits (Alzamora et al., 2010; Gong et al., 2010; Eaton et al., 2021). In A-IC cells, H^+^-ATPase plays an essential role in pumping H^+^ into the lumen of the collecting duct, thus regulating the systemic acid base balance (Soleimani and Rastegar, 2016; Bissler et al., 2019b; Barone et al., 2021; Do et al., 2022; Barone et al., 2023).

In addition to H^+^-ATPase, the electrogenic 2Cl^−^/H^+^ exchanger (CLC5) is also expressed on lysosomal membranes and works in tandem with H^+^-ATPase to maintain the luminal acidity of this crucial organelle in several nephron segments (Günther et al., 1998; Satoh et al., 2017). Both CLC5 and H^+^-ATPase are known to be expressed in A-IC cells (Chen et al., 2017; Park et al., 2018), and show co-localization on both the intracellular organelle membranes and the apical membrane of A-IC cells (Günther et al., 1998; Satoh et al., 2017). In kidneys of various mouse models of TSC (*Tsc1* or *Tsc2* KO in principal cells, *Tsc1* KO in pericytes, as well in *Tsc2* ^+/−^), V H^+^-ATPase and CLC5 show robust expression and remarkable co-localization on the apical membrane of A-IC cells lining the cysts (Barone et al., 2023).

The mode of transport of CLC5 is electrogenic and comprises the inward movement of 2Cl^−^ in exchange for an outward movement of H^+^ from the lysosomal lumen (Günther et al., 1998; Satoh et al., 2017). The co-localization of V H^+^-ATPase and CLC5 in both the lysosomal membrane and the plasma membrane support the view that both molecules may traffic from the intracellular compartments to the plasma membrane in A-IC cells in TSC (Barone et al., 2023). It has been speculated that CLC5 co-localization with H^+^-ATPase on the apical membrane of A-IC cells could provide a mechanism for continued chloride secretion while maintaining the gradient for continuous H^+^-ATPase H^+^ secretion into the cyst lumen (Barone et al., 2023).

## The central role of FOXI1 in kidney cystogenesis in TSC

RNA sequencing (RNA-Seq) and confirmatory expression studies in our laboratories demonstrated robust expression of FOXI1 and its downstream targets, including H^+^-ATPase and cytoplasmic carbonic anhydrase 2 (CAII), in the cyst epithelia of *Tsc1* (or *Tsc2*) knockout (KO) mice, but not in *Pkd1* mutant mice (Barone et al., 2021; Barone et al., 2023). FOXI1 belongs to a large family of transcription factors that are important in cell-type specification during organogenesis (Benayoun et al., 2011). In the kidney, FOXI1 is exclusively expressed in IC cells and is necessary for expression of many H^+^-ATPase subunits in this nephron segment (Al-Awqati and Schwartz, 2004; Blomqvist et al., 2004; Vidarsson et al., 2009). In addition, FOXI1 is likely a major determinant for proper assembly of H^+^-ATPase subcomplexes and its activation at both the plasma membrane and the membranes of intracellular organelles.

Deletion of FOXI1, which is vital to H^+^-ATPase expression and IC cell development (Blomqvist et al., 2004), completely inhibited mTOR activation and abrogated the cyst burden in *Tsc1* KO mice (Barone et al., 2021). These results unequivocally demonstrate the critical role that IC cells, along with their H^+^-ATPase and acid secreting machinery, play in TSC kidney cystogenesis. The collecting ducts of the FOXI1 null mice and the Tsc1/Foxi1 double KO (dKO) mice contained primitive cells that expressed both intercalated cell and principal cell transporters (Blomqvist et al., 2004; Barone et al., 2021). Deletion of FOXI1 resulted in profound suppression of several activated H^+^-ATPase subunits in the IC cells of Tsc1 KO mice (Barone et al., 2021).

Figure 2 is a schematic diagram that depicts the interaction of mTORC1 and H^+^-ATPase on the lysosomal membrane. The left panel depicts the state of mTORC1 in an inactivated state. As noted, the TSC1-TSC2 complex inhibits mTORC1 by negatively regulating RHEB-GTPase. Inactivating mutations in TSC1 or TSC2 remove the inhibitory effect of TSC complex on RHEB-GTPase leading to the activation of mTORC1, which then phosphorylates the downstream elements, S6K, and the eIF4E-binding proteins (4-EBP), enhancing cell proliferation and growth. The feedback loop between mTORC1 and H^+^-ATPase facilitates enhanced expression and assembly of H^+^-ATPase subcomplexes, and its activation by mTORC1. At the same time, the stimulatory effect of H^+^-ATPase on mTORC1 enables its recruitment and activation on lysosomal membrane. Unique to the kidney in TSC is the activation of FOXI1 transcription factor consequent to the activation of mTORC1 (Barone et al., 2021; Barone et al., 2023). FOXI1 is a master regulator of H^+^-ATPase subunits and its inactivation completely abrogated cystogenesis and mTORC1 activation in kidneys of TSC1 KO mice (Barone et al., 2021). As indicated the electrogenic Cl-transporter, CLC-5, is enhanced and translocated to the apical membrane of A-IC cells in addition to its original location on the lysosomal membrane. The CLC-5 maybe involved in movement of Cl- into the cyst lumen and expansion.

## FOXI1-dependent signaling pathways and kidney cystogenesis in TSC

To discern the molecular basis of kidney cystogenesis in *Tsc1* KO mice, we analyzed and contrasted the RNA transcriptomes in: 1) *Tsc1* single KO mice which have numerous cysts and cyst adenomas; and compared it to 2) *Tsc1/Foxi1* dKO mice that do not exhibit any kidney cysts. Our results identified the proto-oncogene c-KIT and the vasopressin receptor 1A (AVPR1A) as robustly enhanced transcripts in kidneys of *Tsc1* KO mice (Zahedi et al., 2022; Zahedi et al., 2023). Both molecules exhibit enhanced expression at the mRNA and protein levels in kidney cysts in TSC1 KO mice. Both transcripts were completely suppressed in *Tsc1/Foxi1* dKO mice (Zahedi et al., 2022; Zahedi et al., 2023). Both molecules are known to be expressed in A-IC cells under baseline conditions (Chen et al., 2017; Park et al., 2018).

Transfection of cultured m-IMCD cells with the full length FOX1 cDNA strongly induced the expression of c-KIT and AVPR1A (Zahedi et al., 2022; Zahedi et al., 2023). These results demonstrate that FOXI1 which is critical to the kidney cystogenesis in TSC (Barone et al., 2021), directly induces the expression of c-KIT and AVPR1A in cystic kidneys. Both genes, c-KIT (Hirota et al., 1998; Wiedmann and Caca, 2005; Larkin and Eisen, 2006; Bosbach et al., 2017; Lasota et al., 2019) and AVPR1A (Miller et al., 2013; Fenner, 2019; Zhao et al., 2019; Heidman et al., 2022), are known to play essential roles in enhancing cell proliferation in several uroepithelial cancers and other malignancies in part via mTORC1 activation. Both c-KIT and AVPR1A exhibit predominant expression in IC cells (Chen et al., 2017), with AVPR1A playing a crucial role in H^+^-ATPase stimulation in IC cells and acid secretion into the lumen of the collecting ducts (Yasuoka et al., 2013; Giesecke et al., 2019). This is in stark contrast with the cyst epithelia in ADPKD, which contain abundant principal cells with very few IC cells (Barone et al., 2021) and do not show any upregulation of c-KIT or AVPR1A (Zahedi et al., 2022; Zahedi et al., 2023).

c-KIT is a receptor tyrosine kinase that is expressed in several cell types and plays a critical role in enhancing cell proliferation and growth (Miettinen et al., 2005; de Toledo et al., 2023; Krimmer et al., 2023; Sternberg et al., 2023). Published reports indicate that increased activity of the kinase domain of c-KIT significantly contributes to the increased incidence of cancer consequent to uncontrolled cell proliferation. Such aberrant activity of c-KIT has been implicated in the pathogenesis of gastrointestinal stromal tumors (GISTs), mastocytosis, and hematological malignancies (Wiedmann and Caca, 2005; Bosbach et al., 2017; Lasota et al., 2019).

In the kidney, AVPR1A plays a key role secreting acid into the lumen of the collecting duct via stimulation of H^+^- secretion in A-IC cells (Yasuoka et al., 2013; Giesecke et al., 2019). Basolateral treatment of isolated perfused medullary collecting ducts with the AVPR1A agonist or vasopressin increased intracellular calcium levels in IC cells, enhanced apical abundance of H^+^-ATPase, and stimulated H^+^ secretion (Yasuoka et al., 2013; Giesecke et al., 2019). AVPR1A is essential in increasing cell proliferation in several tissues including prostate epithelial cells (Miller et al., 2013; Fenner, 2019; Zhao et al., 2019). The treatment of Castration-resistant prostate cancer (CRPC) cells with the AVPR1A ligand, arginine vasopressin (AVP), activated cAMP response element-binding protein (CREB) via the RAS-MAPK transduction cascade (Zhao et al., 2019). Inhibition by the selective AVPR1A antagonist, relcovaptan, decreased CRPC proliferation in mouse models (Miller et al., 2013). In addition, depletion of AVPR1A in CRPC significantly inhibited cell proliferation (Miller et al., 2013; Fenner, 2019).

## Conclusion

The studies that are discussed in this review article demonstrate that in TSC renal cysts, A-IC cells that express both TSC1 and TSC2 constitute the cyst epithelium, and that FOXI1 plays a critical role in the process of cystogenesis. Currently, the only therapeutic option for TSC renal cystic disease is treatment with mTORC1 inhibitors, such as everolimus, which is fraught with many limitations, including resistance to treatment or the return of cysts and tumors to their original size upon discontinuation of therapy. Development of a thorough understanding of the process of renal cystogenesis in TSC should provide us with novel druggable targets for the treatment of this disorder. Delineating specific molecules and pathways that maybe critical to cyst expansion are part of the systemic approach aimed at understanding the mechanistic basis of TSC renal cystogenesis and developing novel and effective therapies for its treatment. The identification of c-KIT and AVPR1A as two differentially expressed genes with known effects on promotion of cell growth and mTORC1 activation in uroepithelial carcinomas and other malignancies is likely a first step toward the exciting discovery of new therapies for this devastating disease.
